# The ALS/FTLD associated protein C9orf72 associates with SMCR8 and WDR41 to regulate the autophagy-lysosome pathway

**DOI:** 10.1186/s40478-016-0324-5

**Published:** 2016-05-18

**Authors:** Peter M. Sullivan, Xiaolai Zhou, Adam M. Robins, Daniel H. Paushter, Dongsung Kim, Marcus B. Smolka, Fenghua Hu

**Affiliations:** Department of Molecular Biology and Genetics, Weill Institute for Cell and Molecular Biology, Cornell University, 345 Weill Hall, Ithaca, NY 14853 USA

**Keywords:** Frontotemporal lobar degeneration, Amyotrophic lateral sclerosis, Autophagy, Lysosome, C9orf72, SMCR8, WDR41, Ulk1, FIP200/RB1CC1

## Abstract

Hexanucleotide repeat expansion in the C9orf72 gene is a leading cause of frontotemporal lobar degeneration (FTLD) with amyotrophic lateral sclerosis (ALS). Reduced expression of C9orf72 has been proposed as a possible disease mechanism. However, the cellular function of C9orf72 remains to be characterized. Here we report the identification of two binding partners of C9orf72: SMCR8 and WDR41. We show that WDR41 interacts with the C9orf72/SMCR8 heterodimer and WDR41 is tightly associated with the Golgi complex. We further demonstrate that C9orf72/SMCR8/WDR41 associates with the FIP200/Ulk1 complex, which is essential for autophagy initiation. C9orf72 deficient mice, generated using the CRISPR/Cas9 system, show severe inflammation in multiple organs, including lymph node, spleen and liver. Lymph node enlargement and severe splenomegaly are accompanied with macrophage infiltration. Increased levels of autophagy and lysosomal proteins and autophagy defects were detected in both the spleen and liver of C9orf72 deficient mice, supporting an in vivo role of C9orf72 in regulating the autophagy/lysosome pathway. In summary, our study elucidates potential physiological functions of C9orf72 and disease mechanisms of ALS/FTLD.

**Electronic supplementary material:**

The online version of this article (doi:10.1186/s40478-016-0324-5) contains supplementary material, which is available to authorized users.

## Introduction

Frontotemporal lobar degeneration (FTLD) and amyotrophic lateral sclerosis (ALS) are two devastating neurodegenerative diseases, which due to overlaps in clinical presentations, pathological features, and genetic causes, are considered two manifestations of a continuous disease spectrum [1, 7, 15, 29, 30, 49].

Many novel genes have been recently associated with ALS/FTLD [29, 37]. Among these, hexanucleotide repeat expansion in the C9orf72 gene has been shown to be the main cause of ALS/FTLD [13, 38, 51], for which three disease mechanisms have been proposed: toxicity of RNA foci formed by RNA repeats, toxicity induced by dipeptide repeat aggregation as a result of repeat associated non-ATG mediated RNA translation (RAN), and reduced expression of the C9orf72 gene [26, 39]. Recently, several animal models have been established to investigate repeat associated gain of toxicity [10, 18, 31, 33, 35, 47] as well as the physiological functions of C9orf72 in mammals. While neuron and glia specific C9orf72 ablation or intracerebral mRNA knockdown does not seem to cause motor neuron disease in mouse models [22, 24], two recent studies on whole body C9orf72 deficient mice demonstrate that C9orf72 deficiency results in severe immune dysregulation [2, 34], suggesting that loss of C9orf72 function could lead to mis-regulated inflammatory responses.

Despite many attempts at characterizing the exact molecular function of C9orf72, its cellular role is still not entirely clear. Bioinformatics studies have predicted C9orf72 to be a member of DENN domain containing proteins which typically function as guanine exchange factors for Rab GTPases, key regulators of membrane trafficking in eukaryotic cells [28, 54]. One study has demonstrated physical interactions between C9orf72 and Rab7 and Rab11, GTPases involved in late endosome maturation or endosome recycling, respectively, and a role of C9orf72 in autophagy regulation [14]. However, direct regulation of these Rab GTPases by C9orf72 was not demonstrated in this study [14].

In order to gain insight into C9orf72 function, we performed a proteomic screen for C9orf72 binding partners. We showed that C9orf72 forms a tight complex with SMCR8, another DENN domain containing protein [54], and WDR41, a WD40 repeat protein. We further demonstrated the physical interaction between C9orf72/SMCR8/WDR41 and the FIP200/Ulk1/ATG13/ATG101 complex, which is an essential regulator of autophagy initiation. Our results are consistent with two recent reports on the C9orf72/SMCR8/WDR41 interaction published while this manuscript was under preparation [41, 53]. Furthermore, our data from C9orf72 deficient mice support a role of C9orf72 in immune regulation and the autophagy-lysosome pathway.

## Material and methods

### DNA and Plasmids

Human C9orf72 (C9-L), WDR41 and SMCR8 cDNAs were from the human ORFome 8.1 library and cloned into pQCXIN, pEGFP-N1 or pEGFP-N2, respectively, with an N-terminal (C9orf72) or C-terminal (WDR41 and SMCR8) GFP tag. The short C9orf72 isoform was cloned from C9-L into pEGFP-C1 with the following 3' primer: tgacCTCGAGttacttgagaagaaagccttcatg. WDR41 and SMCR8 were also cloned into pcDNA3.1 myc his A (Invitrogen) to generate C-terminal myc his tagged constructs. Additionally, C9orf72 and WDR41 were cloned into p3xFLAG-CMV7.1 (Sigma) to generate N-terminal FLAG tagged constructs. Myc-ATG13, FLAG-ATG101 and p3xFLAG-CMV10-hFIP200 were obtained from Addgene (plasmid #31965, 22877 and 24300, respectively).

### Pharmacological Reagents and Antibodies

The following primary antibodies were used in this study: anti-FLAG (M2) and anti-myc (9E10) from Sigma-Aldrich, anti-GAPDH from Abcam (ab8245), anti-LC3B (GTX127375) and anti-C9orf72 (GTX119776) from GeneTex, anti-Cathepsin D (sc-6486), anti-Ulk1 (sc-33182) and anti-C9orf72 (sc-138763) from Santa Cruz Biotechnology, anti-C9orf72 (AP12928b) and anti-FIP200 (17250-1-AP) from Proteintech, anti-SMCR8 from Bethyl Laboratories, anti-WDR41 from Abgent, rat anti-CD68 from AbD Serotec, sheep anti-progranulin from R&D systems and anti-mouse LAMP1 (553792) from BD Biosciences. Anti-mouse prosaposin antibody was generated by Pocono Rabbit Farm and Laboratory and was previously characterized [55]. Anti-C9orf72-long isoform [52] was a gift from Dr. Janice Robertson (University of Toronto); anti-GFP antibody was a gift from Dr. Anthony Bretscher (Cornell University); and anti-GPP130 was a gift from Dr. William Brown (Cornell University). The following secondary antibodies were used: donkey anti-mouse 800 and donkey anti-rabbit 800 from LI-COR, AlexaFluor donkey anti-goat 680, donkey anti-rabbit 680, donkey anti-mouse 680, and donkey anti-rat 680 from Invitrogen, and donkey anti-mouse 568 from Biotium. Hoechst stain was obtained from Invitrogen. Brefeldin A (BFA) and nocodazole were obtained from Sigma-Aldrich and used at a final concentration of 300 μM and 20 mM, respectively.

### Mouse strains

C9orf72 knockout mice were produced using CRISPR/Cas9 genome editing with a guide RNA (gRNA) targeting exon 2 of mouse gene 3110043O21RIK. C57BL/6 J x FvB/N mouse embryos were injected with gRNA and Cas9 mRNA at the Cornell Transgenic Core Facility. Editing was confirmed by sequencing PCR products from genomic DNA and loss of protein products was determined by Western blot of tissue lysate. Offspring from the founder containing 1 bp deletion were used for the study except the 10 month old founder mice. The following primers were used to genotype C9orf72 knockout mice: 5′- gcggctacctttgcttac -3′ (WT forward), 5′- tggcggctacctttgcta -3 (KO forward) and 5′- tgcccaggagacacaacata -3′ (common reverse).

### Cell culture and DNA transfection

HEK293T cells were maintained in Dulbecco’s Modified Eagle’s Medium (Cellgro) supplemented with 10 % fetal bovine serum (Gibco) and 1 % Penicillin–Streptomycin (Invitrogen) in a humidified incubator at 37 °C and 5 % CO_2_. Cells were transiently transfected with polyethyleneimine as described [48].

### Immunoprecipitation and protein analysis

Cells were lysed in 50 mM Tris pH 8.0, 150 mM NaCl, 1 % Triton X-100, and 0.1 % deoxycholic acid with protease inhibitors (Roche). Lysates were incubated with GFP-Trap beads (ChromoTek) or anti-FLAG antibody conjugated beads (Sigma-Aldrich) for 2–3 h at 4 °C. Beads were washed 3 times with 50 mM Tris pH 8.0, 150 mM NaCl, and 1 % Triton X-100. Samples were denatured in 2xSDS sample buffer (4 % SDS, 20 % glycerol, 100 mM Tris pH 6.8, 0.2 g/L bromophenol blue) by boiling for 3 min. Samples were run on 8 % or 12 % polyacrylamide gels and transferred to PVDF membranes (Millipore). Membranes were blocked in either Odyssey Blocking Buffer (LI-COR Biosciences) or 5 % non-fat milk in PBS for 1 h followed by incubation with primary antibodies overnight at 4 °C. Membranes were washed 3 times with Tris-buffered saline with 0.1 % Tween-20 (TBST) then incubated with secondary antibody for 2 h at room temperature. Membranes were washed 3 times with TBST and imaged using an Odyssey Infrared Imaging System (LI-COR Biosciences).

To quantify protein levels in tissue samples, tissues were homogenized in RIPA buffer (50 mM Tris pH 8.0, 150 mM NaCl, 1 % Triton X-100, 0.1 % SDS and 0.1 % deoxycholic acid) with protease inhibitors on ice and then equal volume of 2X SDS sample buffer was added before sonication. 50 μg of each protein sample was loaded onto a 12 % poly-acrylamide gel. Blots were analyzed by LiCor Odyssey system and normalized to GAPDH.

### SILAC and mass spectrometry analysis

N2a cells were grown a minimum of five generations in DMEM with 10 % dialyzed FBS (Sigma) supplemented with either light (C12, N14 arginine and lysine) or heavy (C13, N15 arginine and lysine) amino acids. The heavy cells were transfected in two 15 cm dishes with GFP-C9orf72 expression constructs while the light cells were transfected with pEGFP-C1 as a control. Two days after transfection, cells were lysed in 50 mM Tris pH8.0, 150 mM NaCl, 1 % Triton, 0.1 % deoxycholic acid with protease inhibitors (Roche). The lysates were subject to anti-GFP immunoprecipitation using GFP-Trap beads (ChromoTek). The presence of GFP and GFP-C9orf72 in immunoprecipitated samples was confirmed by SDS-PAGE and Krypton staining (Invitrogen). Samples were then combined and boiled 5 min with 1 % DTT followed by alkylation by treating samples with a final concentration of 28 mM iodoacetamide. Proteins were precipitated on ice for 30 min with a mixture of 50 % acetone/49.9 % ethanol/0.1 % acetic acid. Proteins were pelleted and washed with this buffer, re-precipitated on ice, and dissolved in 8 M urea/50 mM Tris pH 8.0 followed by dilution with three volumes of 50 mM Tris pH 8.0/150 mM NaCl. Proteins were digested overnight at 37 ° C with 1 μg mass-spec grade Trypsin (Promega). The resulting peptide samples were cleaned up for mass spectrometry by treatment with 10 % formic acid and 10 % trifluoroacetic acid (TFA) and washed twice with 0.1 % acetic acid on pre-equilibrated Sep-Pak C18 cartridges (Waters). Samples were eluted with 80 % acetonitrile (ACN)/0.1 % acetic acid into silanized vials (National Scientific) and evaporated using a SpeedVac. Samples were re-dissolved in H2O with ~1 % formic acid and 70 % ACN. Peptides were separated using hydrophilic interaction liquid chromatography (HILIC) on an Ultimate 300 LC (Dionex). Each fraction was evaporated with a SpeedVac and resuspended in 0.1 % TFA with 0.1 pM angiotensin internal standard. Samples were run on a Thermo LTQ Orbitrap XL mass spectrometer and data analyzed using the SORCERER system (Sage-N research).

### Hematoxylin and eosin (H&E) staining

Mouse tissues were fixed with 4 % formaldehyde. After dehydration with 70 % ethanol, tissues were embedded with paraffin. The tissues were sliced to 8 μm. Followed by deparaffiinization with xylene and ethanol (100 %, 95 %, 80 %) and rehydration with tap water, the slides were stained in hematoxalin for 3 min, destained with acid ethanol and rinsed with tap water, and then stained with eosin for 30 s. The slides were then dehydrated with ethanol and xylene and mounted.

### Immunofluorescence microscopy

HeLa cells grown on glass coverslips were fixed in 3.7 % paraformaldehyde for 15 min, washed 3 times with PBS, and permeabilized and blocked in Odyssey Blocking Buffer with 0.05 % saponin or 0.1 % Triton for 20 min. Primary antibodies diluted in blocking buffer with 0.05 % saponin were applied to the cells overnight at 4 °C. Coverslips were washed 3 times with PBS. Secondary antibodies and Hoechst stain diluted in blocking buffer with 0.05 % saponin were applied to the cells for 2 h at room temperature. Coverslips were washed and mounted onto slides with Fluoromount G (Southern Biotech). Images were acquired on a CSU-X spinning disc confocal microscope (Intelligent Imaging Innovations) with an HQ2 CCD camera (Photometrics) using a 100x objective.

Mouse tissues were perfused and fixed with 4 % formaldehyde. After gradient dehydration with 15 % and 30 % sucrose, tissues were embedded with OCT compound (Sakura Finetek USA) and sectioned with Cryotome. For the immunostaining, tissue sections were permeabilized and blocked in Odyssey blocking buffer with 0.05 % saponin for 1 h. Primary antibodies were incubated in blocking buffer overnight at 4 °C. Sections were washed and incubated in secondary antibodies conjugated to Alexaflour 488, 568, or 660 (Invitrogen). Sections were washed three more times and coverslips mounted onto slides with Fluoromount G (Southern Biotech). Images were acquired on a CSU-X spinning disc confocal microscope (Intelligent Imaging Innovations) with an HQ2 CCD camera (Photometrics) using a 40x objective.

### Statistical analysis

The data were presented as mean ± SEM. Two-group analysis was performed using the Student’s *t* test. *P*-values <0.05 were considered statistically significant.

## Results

### C9orf72 forms a complex with SMCR8 and WDR41

**Fig. 1:**
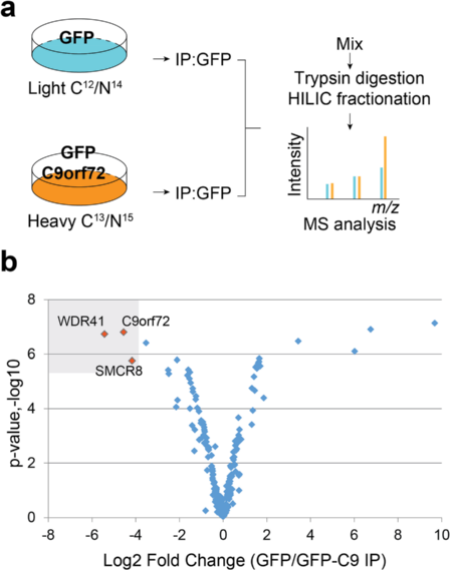
SILAC proteomic screen for C9orf72 binding partners. **a** Schematic workflow of SILAC proteomic screen used to identify C9orf72 protein interactions. **b** Volcano plot of SILAC hits. Hits with more than 10 peptides are plotted. Top hits identified in the heavy fraction are highlighted

**Fig. 2:**
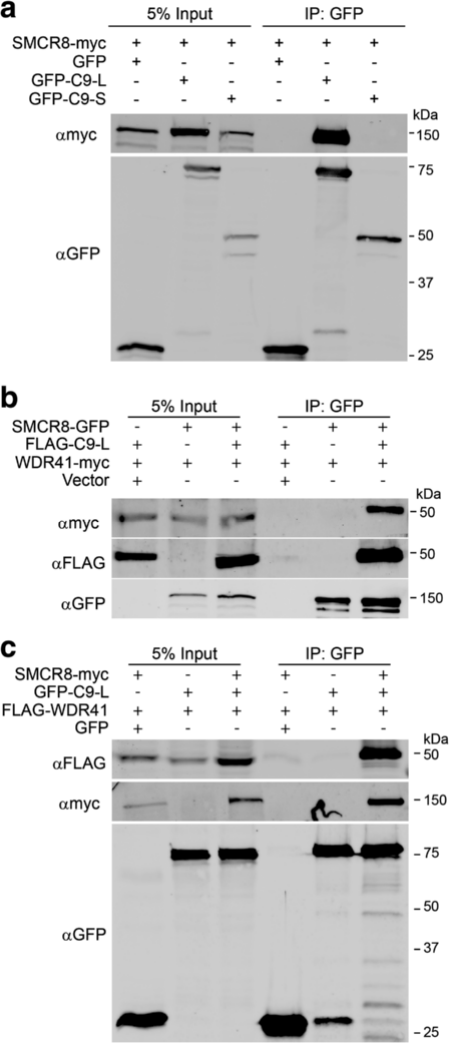
Co-immunoprecipitation between C9orf72, SMCR8 and WDR41. **a** GFP-tagged human C9orf72 isoform I (GFP-C9-L) or isoform II (GFP-C9-S) were overexpressed with SMCR8-myc in HEK293T cells and immunoprecipitated by anti-GFP beads. **b** SMCR8-GFP and WDR41-myc were coexpressed with or without FLAG-C9-L and the lysates were immunoprecipitated using anti-GFP antibodies. **c** GFP-C9-L and FLAG-WDR41 were co-expressed with or without SMCR8-myc as indicated and the lysates were immunoprecipitated using anti-GFP antibodies

In humans, two C9orf72 protein isoforms are generated from three alternatively spliced transcripts, a long form (C9-L) and a short form (C9-S), with multiple studies showing that the protein and mRNA level of the C9-L form are decreased in C9/ALS patients [5, 50, 52]. To decipher the protein interaction network of C9orf72, a SILAC (stable isotope labeling of amino acids in cell culture) based proteomic screen was performed in the neuroblastoma cells line neuro-2a (N2a) using GFP-C9orf72 (C9-L) as the bait and GFP as a control (Fig. 1a, Additional file 1: Figure S1). Several proteins were found to be enriched in the C9orf72 immunoprecipitations (IPs) (Fig. 1b, Additional file 2: Table S1). The top two hits from the screen were SMCR8 and WDR41, two proteins of unknown functions (Fig. 1b). Interestingly, like C9orf72, SMCR8 was also predicted to contain a DENN domain [54]. The interaction between C9orf72, SMCR8, and WDR41 was verified using co-IPs in transfected HEK293T cells (Fig. 2a-c). SMCR8 strongly interacts with GFP-C9orf72 but not GFP in the co-IP experiment (Fig. 2a). Moreover, co-expression of C9orf72 consistently increases the level of SMCR8, suggesting that C9orf72 might stabilize overexpressed SMCR8. However, the short isoform of human C9orf72 (C9-S) does not bind SMCR8 (Fig. 2a). Thus we focus on the C9-L form for the rest of the study (hereafter referred to as C9orf72). While we failed to detect any interaction between WDR41 and C9orf72 or SMCR8 when WDR41 is expressed with either C9orf72 or SMCR8 alone, WDR41 strongly co-immunoprecipitates with C9orf72 and SMCR8 when C9orf72 and SMCR8 are co-expressed, suggesting that WDR41 interacts only with the C9orf72/SMCR8 heterodimer (Fig. 2b, 2c).

### Cellular localization of C9orf72, SMCR8 and WDR41

**Fig. 3:**
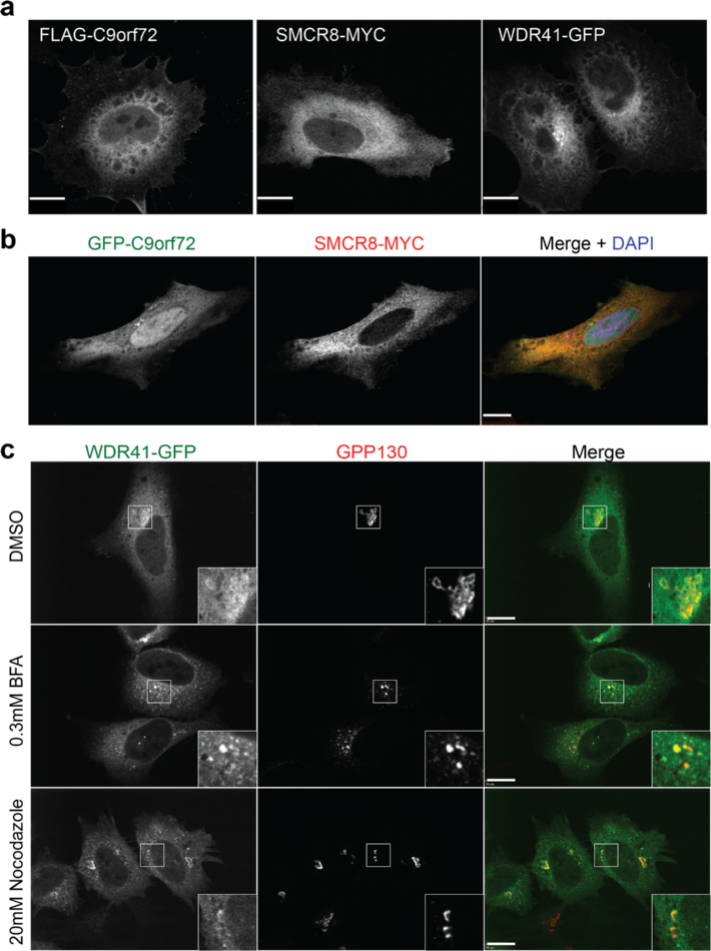
Cellular localization of C9orf72, SMCR8 and WDR41. **a** HeLa cells were transfected with FLAG-C9orf72 (C9-L), SMCR8-myc or WDR41-GFP. Cells were stained with anti-FLAG or anti-myc to visualize FLAG-C9orf72 or SMCR8-myc, respectively. Maximum projection images from confocal sections are shown. Scale bar = 10 μm. **b** HeLa cells were transfected with GFP-C9orf72 (C9-L) and SMCR8-myc. Cells were stained with anti-myc antibodies to visualize SMCR8-myc. **c** WDR41-GFP expressing HeLa cells were treated with DMSO control, 0.3 mM BrefeldinA (BFA), or 20 mM Nocodazole for 2 h. Cells were stained with anti-GPP130 antibodies to label cis-Golgi. Single confocal images are shown for b and c. Scale bar = 10 μm

To gain insight into the cellular function of the C9orf72/SMCR8/WDR41 complex, we expressed these proteins in HeLa cells and examined their distribution within the cell. Both C9orf72 and SMCR8 show diffuse cytoplasmic localization, when expressed alone or together (Fig. 3a and b). Nuclear localization was observed for C9orf72, especially the GFP-tagged C9orf72 but not SMCR8 (Fig. 3a and b). WDR41 also shows diffuse cytoplasmic distribution (Fig. 3a and c). However, careful examination reveals enrichment of WDR41 at the cis-Golgi, which is confirmed when labelled by the cis-Golgi protein GPP130 (Fig. 3c). This is further supported by treatment of cells with BrefeldinA, which causes the Golgi to collapse. Even after such treatment, WDR41 remains colocalized with GPP130, indicating that WDR41 is tightly associated with the Golgi membrane. Similar results were obtained after treatment with nocodazole, a microtubule destabilizing drug that causes the Golgi to disperse (Fig. 3c).

### Interaction of C9orf72/SMCR8/WDR41 with the FIP200 autophagy initiation complex

**Fig. 4:**
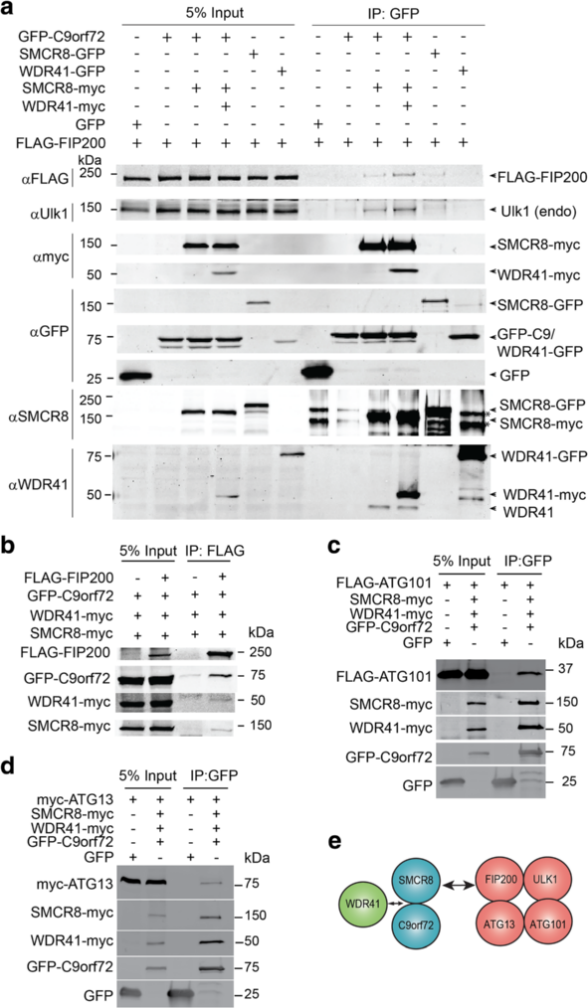
The C9orf72/SMCR8/WDR41 complex interacts with FIP200/Ulk1. **a** Co-immunoprecipitation between C9orf72/SMCR8/WDR41 and FIP200 and Ulk1. HEK293T cells were transfected with FLAG-FIP200 and C9orf72, SMCR8 and/or WDR41 as indicated. Cells were lysed 40 h after transfection and lysates were immunoprecipated with anti-GFP antibodies. Lysates and immunoprecipitates were analyzed by Western blots as indicated. * indicated non-specific bands recognized by anti SMCR8 antibodies in the IP products. Representative images from 3 independent experiments are shown. **b** Co-immunoprecipitation between C9orf72/SMCR8/WDR41 and FIP200. HEK293T cells were transfected as indicated and lysed and immunoprecipitated using anti-FLAG antibodies. Lysates and immunoprecipitates were analyzed by Western blots. **c**, **d** Co-immunoprecipitation between C9orf72/SMCR8/WDR41 and ATG101 (c) or ATG13 (d). HEK293T cells were transfected as indicated and lysed and immunoprecipitated using anti-GFP antibodies. Lysates and immunoprecipitates were analyzed by Western blots. **e** Schematic drawing of the interaction between C9orf72/SMCR8/WDR41 and the FIP200/Ulk1 complex. WDR41 interacts with the C9orf72/SMCR8 dimer to form ternary complex, which then interacts with the FIP200/Ulk1/ATG13/ATG101 complex

A previously reported proteomics screen identified SMCR8 as a binding partner for FIP200 (also called RB1CC1) [4], a protein involved in autophagy initiation [44]. However, the interaction between SMCR8 and FIP200 is barely detectable by co-IP when only these two proteins are overexpressed (Fig. 4a). Interestingly, we found that co-transfection of C9orf72 and SMCR8 allows much stronger binding of SMCR8 to FIP200, which is further enhanced by WDR41 overexpression, suggesting that C9orf72/SMCR8/WDR41 forms a ternary complex to interact with FIP200 (Fig. 4a). The weaker binding between SMCR8 and FIP200 when SMCR8 is expressed alone in HEK293T cells is most likely explained by very low endogenous levels of C9orf72 and WDR41 (Fig. 4a and Additional file 1: Figure S1). Ulk1, a kinase that interacts with FIP200 in the autophagy initiation complex [44], binds the C9orf72/SMCR8/WDR41 in a similar manner as FIP200 (Fig. 4a). Furthermore, C9orf72, SMCR8 and WDR41 are all detected in the FIP200 immunoprecipitates (Fig. 4b). ATG13 and ATG101, two other proteins associated with FIP200/Ulk1 also interact with C9orf72/SMCR8/WDR41 (Fig. 4c and d). These data support that the formation of the C9orf72/SMCR8/WDR41 complex allows their interaction with the FIP200/Ulk1/ATG13/ATG101 complex (Fig. 4e).

### Generation and characterization of C9orf72 deficient mice

**Fig. 5:**
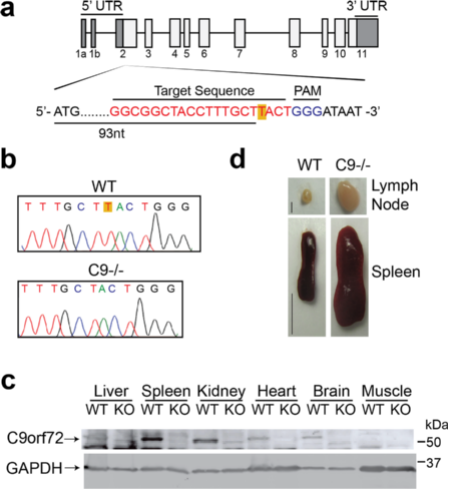
Generation of C9orf72 deficient mice. **a** Schematic drawing of the mouse homologue of C9orf72, 3110043O21RIK, and the site targeted for editing by CRISPR/Cas9. **b** Sequencing traces of wildtype (top) and edited (bottom) C9orf72 from genomic PCR show a one nucleotide deletion (highlighted with yellow) near the Cas9 cleavage site. **c** Western blot analysis of C9orf72 protein levels in wild type (WT) and C9orf72-/- (KO) mouse tissues with anti-C9-L antibodies. **d** Representative images of cervical lymph nodes and spleen from 4-5 months old WT and C9orf72-/- mice. Scale bar = 2 mm (lymph node); 1 cm (spleen)

Three protein isoforms of mouse C9orf72 homolog (I, 55 kDa; II, 35 kDa and III; 50 kDa) have been identified with isoform I (55 kDa), the functional homolog of C9-L in humans, being the dominant form in most tissues [3, 45]. In order to investigate the function of C9orf72 in vivo, we generated C9orf72 deficient mice using the CRISPR/Cas9 system [12, 32]. Guide RNA targeted close to the start codon of the mouse C9orf72 isoform I and III was co-injected with the Cas9 mRNA into the pronuclei of fertilized eggs (Fig. 5a). All the offspring displayed Cas9 mediated cleavage and editing from non-homologous end joining mediated repair. We chose one founder containing a one-nucleotide deletion at the beginning of the C9orf72 open reading frame, resulting in a frame shift at residue 33 that produces a stop codon at amino acid 40 (Fig. 5b). Based on the splicing patterns, the one nucleotide deletion should cause the loss of protein products for C9orf72 isoforms I (55 kDa) and III (50 kDa), but should not affect isoform II (35 kDa). Indeed, Western blot using several antibodies shows loss of C9orf72 isoform I protein in homozygous offspring from the above-mentioned founder (Fig. 5c, and Additional file 3: Figure S2). We failed to detect isoform II and III in our Western blots with all the C9orf72 antibodies tested and thus we cannot determine whether these isoforms are affected by the CRISPR/Cas9 editing. While C9orf72 isoform I is detectable in all tissues examined, its level is highest in spleen, followed by kidney, brain, and heart, and much lower in the liver and muscle (Fig. 5c). This expression pattern is consistent with a recent publication demonstrating the highest levels of C9orf72 in CD11b^+^ myeloid cells [34].

Our C9orf72 deficient mice do not have any apparent growth defects (data not shown) but display an obvious lymph node and spleen enlargement phenotype (Fig. 5d), consistent with recent reports on another two independent lines of C9orf72 knockout mice [2, 34]. While occasional mild splenomegaly was observed in the 2 month old C9orf72 deficient mice, this phenotype becomes more severe with age, with the 4-5 month old C9orf72 deficient mouse having a spleen 2-3 times the size of its littermate control (Fig. 5d and Additional file 4: Figure S3). This is also seen in two of the 10 month old founder mice from the CRISPR mediated editing (Additional file 4: Figure S3). The liver also shows slight enlargement but there are no obvious gross anatomical defects in the brain of C9orf72 deficient mice (data not shown).

**Fig. 6:**
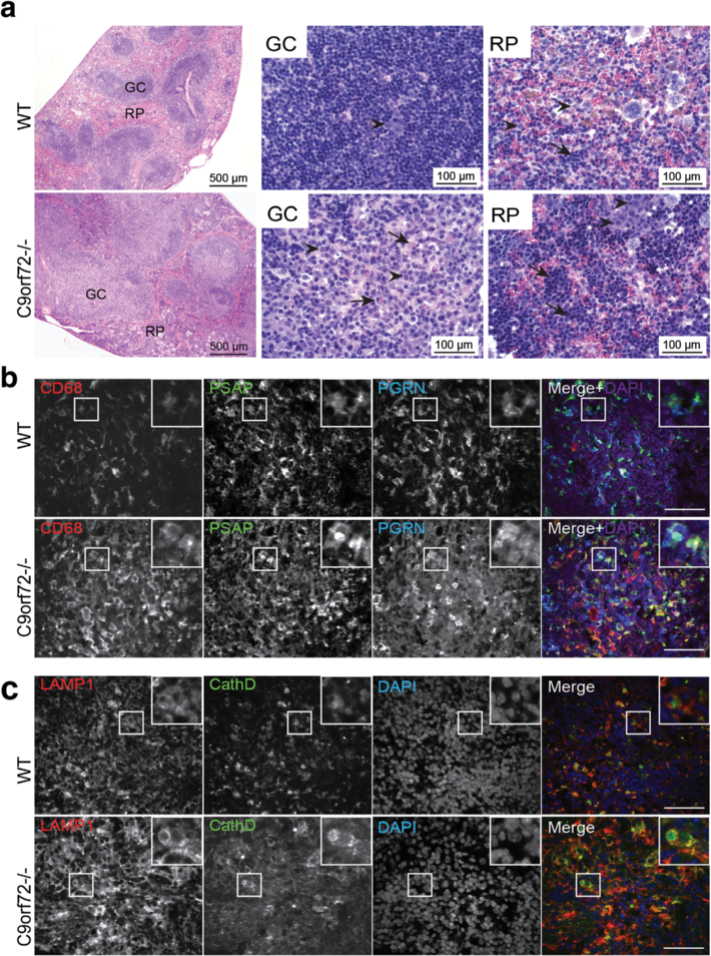
C9 deficiency in mice results in an enlarged spleen phenotype and macrophage infiltration into the spleen. **a** H&E staining of spleen tissues from 5 month old of WT or C9orf72 -/- mouse with zoomed images of germinal center (GC) and red pulp (RP). (GC): Arrows indicate plasma cells and the arrowheads indicate immature immune cells). (RP): arrowheads indicate myeloid precursors; arrows indicate erythroid precursors. Scale bar = 500 μm (100 μm in the zoomed in images for GC and RP). **b** Immunostaining of 4 month old spleen sections (red pulp region) of WT and C9orf72-/- mice with anti-mouse CD68, prosaposin (PSAP), and progranulin (PGRN) antibodies. Nuclei are labelled with DAPI. Insert shows representative CD68+ macrophage cells. Representative pictures from three pairs of mice are shown. Scale bar = 40 μm. **c** Immunostaining of 4 month old spleen sections (red pulp region) of WT and C9orf72-/- mice with anti-mouse Lamp1, and cathepsin D (CathD) antibodies. Nuclei are labelled with DAPI. Insert shows representative cells. Representative pictures from three pairs of mice are shown. Scale bar = 40 μm

**Fig. 7:**
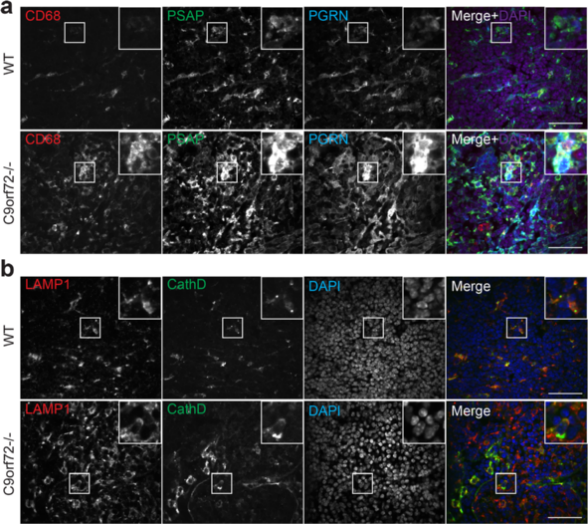
Increased macrophage infiltration and lysosomal proteins in the cervical lymph node of C9 deficient mice. **a** Immunostaining of 4 month old cervical lymph nodes sections (peripheral region) of WT and C9orf72-/- mice with anti-mouse CD68, prosaposin (PSAP), and progranulin (PGRN) antibodies. Nuclei is labelled with DAPI. Insert shows representative CD68+ macrophage cells. Representative pictures from three pairs of mice are shown. Scale bar = 40 μm. **b** Immunostaining of 4 month old cervical lymph nodes sections (peripheral region) of WT and C9orf72-/- mice with anti-mouse Lamp1, and cathepsin D (CathD) antibodies. Nuclei are labelled with DAPI. Insert shows representative cells. Representative pictures from three pairs of mice are shown. Scale bar = 40 μm

**Fig. 8:**
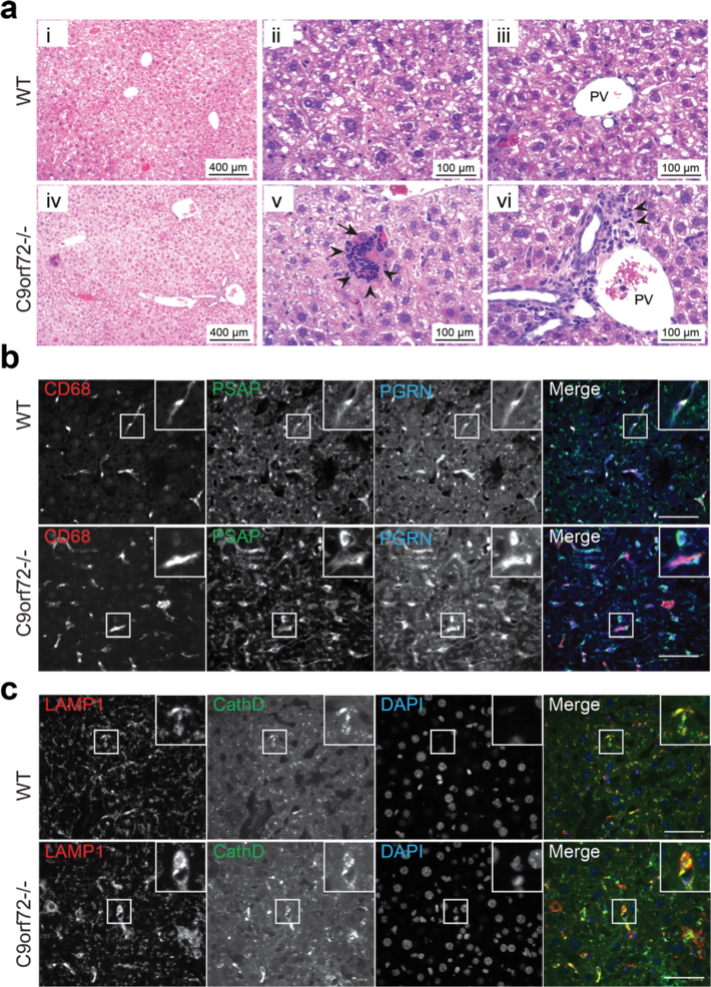
C9 deficiency in mice results in macrophage infiltration and increased levels of lysosomal proteins in the liver. **a** H&E staining of liver tissues from 10 month old of WT or C9orf72 -/- mouse. (ii) and (iii) are high power magnification images of the hepatic parenchyma, arrowhead indicates infiltrated immune cells; arrow points to necrotic hepatocyte. (v) and (vi) are high power magnification images of the hepatic portal area, arrowhead indicates infiltrated immune cells. PV, portal vein. Scale bar: 500 μm in (i) and (iv), 100 μm in (ii, iii, v, vi) **b** Immunostaining of 4 month old liver sections of WT and C9orf72-/- mice with anti-mouse CD68, prosaposin (PSAP), and progranulin (PGRN) antibodies. Nuclei are labelled with DAPI. Insert shows representative CD68+ macrophage cells. Representative pictures from three pairs of mice are shown. Scale bar = 40 μm. **c** Immunostaining of 4 month old liver sections of WT and C9orf72-/- mice with anti-mouse Lamp1, and cathepsin D (CathD) antibodies. Nuclei are labelled with DAPI. Insert shows representative cells. Representative pictures from three pairs of mice are shown. Scale bar = 40 μm

**Fig. 9:**
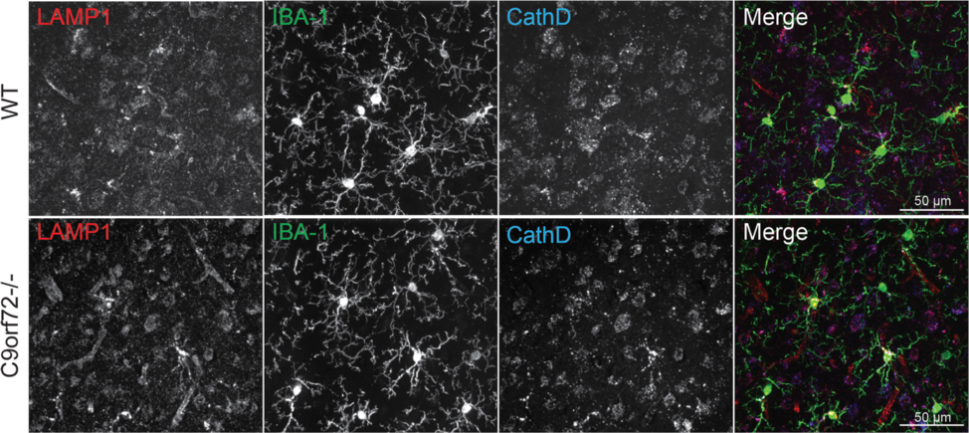
Microglia do not show any obvious abnormalities in C9orf72 deficient mice. Brain sections from 10 month old WT and C9orf72-/- mice were stained with anti-LAMP1, Iba1 and cathepsin D antibodies. Similar results were seen with 5 month old C9orf72-/- mouse. Scale bar = 50 μm

Hematoxylin and eosin (H&E) staining of the C9orf72 deficient spleen reveals enlarged germinal centers (GCs) and abundant extramedullary hematopoietic cells (Fig. 6a). High magnification shows the enlarged GCs are packed with immature immune cells, among which plasma cells are often observed (Fig. 6a). Several types of hematopoietic cells are enriched in the red pulp (Fig. 6a). Immunostaining with anti-CD68 antibodies shows macrophage accumulation or infiltration in the red pulp of the spleen (Fig. 6b), which is also seen in the peripheral regions of cervical lymph nodes (Fig. 7a) and to a lesser extent, liver of C9orf72 deficient mice at 4 months of age (Fig. 8b). In C9orf72-/- liver tissues, infiltrated immune cells were observed in both hepatic portal area and hepatic parenchyma (Fig. 8a). Surrounding by the infiltrated immune cells, necrotic hepatocytes are occasionally seen in C9orf72-/- liver tissues (Fig. 8a). Despite macrophage infiltration in multiple peripheral organs, microglia, the counterpart of macrophages in the central nervous system, do not show any obvious changes in number and morphology in the C9orf72 deficient mice (Fig. 9).

### Autophagy and lysosome defects in the C9orf72 deficient mice

**Fig. 10:**
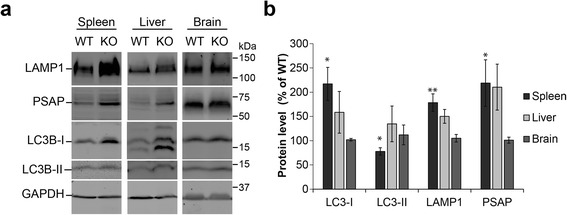
C9 deficiency in mice leads to increased levels of autophagy-lysosome proteins in the spleen. **a** Western blot analysis of tissue lysates from spleen, liver, and brain of WT and knockout mice. Representative pairs are shown. **b** Quantification of autophagy and lysosomal protein levels in knockout mice relative to WT controls. Data are presented as Mean ± SEM, n = 3–6. *, *p* < 0.05; **, *p* < 0.01

Because C9orf72/SMCR8/WDR41 co-immunoprecipitates with the FIP200/Ulk1/ATG13/ATG101 complex, we investigated possible autophagy/lysosome defects in the C9orf72 deficient mice. Interestingly, Ulk1 deficiency has also been reported to result in splenomegaly [23]. Western blot analysis showed significantly increased levels of several proteins involved in the autophagy/lysosomal pathway in spleen and liver lysates of the 2 month old C9orf72 deficient mice compared to littermate controls, including LC3-I, LAMP1, and prosaposin (PSAP), even before the appearance of an obvious splenomegaly phenotype, suggesting that autophagy/lysosome defects might precede anatomical spleen abnormalities (Fig. 10a, b). Although no obvious changes in lysosomal morphology were seen in the C9orf72-/- macrophages, increased levels of the lysosomal proteins progranulin (PGRN), PSAP, LAMP1 and cathepsin D (CathD) are seen in CD68 positive macrophages in the lymph nodes, spleen and liver of C9orf72 deficient mice (Fig. 6, 7 and 8). This might suggest that loss of C9orf72 causes a defect in the lysosome that requires upregulation of lysosomal proteins to compensate. Conversion of LC3-I to LC3-II is an indicator of autophagy initiation [21]. Although LC3-I levels are dramatically increased in C9orf72 deficient spleen lysates, LC3-II levels are significantly reduced (Fig. 10a, b), suggesting that loss of C9orf72 causes a defect in autophagy initiation. Despite these changes in multiple peripheral tissues, brain lysates do not show any apparent increases in autophagy or lysosomal proteins in the 2 month old C9orf72 deficient mice compared to controls (Fig. 10a, b).

## Discussion

The cellular function of C9orf72 has been under intensive investigation since hexanucleotide repeat expansion in the C9orf72 gene was shown to be the main cause of ALS/FTLD [13, 38, 51], with reduced C9orf72 expression proposed as one of the disease mechanisms [39]. In this study, we searched for protein interactors for C9orf72 and identified SMCR8 and WDR41 as two binding partners of C9orf72, consistent with two recently published reports [41, 53]. We further demonstrated that C9orf72/SMCR8/WDR41 interacts with the FIP200/Ulk1/ATG13/ATG101 complex involved in autophagy initiation. Our data also showed that C9orf72 deficient mice display defects in autophagy/lysosome pathway, supporting a critical role of C9orf72 in autophagy/lysosome regulation.

Many other genes involved in membrane trafficking and autophagy have been associated with ALS/FTLD, including TBK1, OPTN, SQSTM1/p62, UBQLN2, VCP/p97 and CHMP2B [19, 27, 36, 40]. Progranulin, the gene mutated in a vast majority of FTLD with ubiquitin positive TDP-43 aggregates, was recently shown to play a critical role in regulating lysosomal function [8, 42] and the progranulin protein was shown to reside in the lysosome [17, 55]. TMEM106B, a risk factor for FTLD with progranulin mutations, also regulates lysosomal morphology and function [6, 9, 20, 25, 43]. Our current results further support that dysfunction in the autophagy lysosome pathway is implicated in the disease progression of ALS/FTLD caused by C9orf72 mutations. C9orf72 deficiency in C. elegans and zebrafish has been shown to result in locomotion defects [11, 46], supporting the notion that C9orf72 haploinsufficiency could contribute to ALS/FTLD progression. While neuron and glia specific C9orf72 ablation or intracerebral mRNA knockdown does not seem to cause motor neuron disease in mouse models [22, 24], our data and recent data by others consistently demonstrate that whole body C9orf72 deficiency produces severe immune dysregulation in mice [2, 34]. In one study, lysosomal abnormalities were also observed in microglia isolated from C9orf72 deficient mice and in the motor cortex and spinal cord of ALS patients with C9orf72 mutations [34]. These findings strongly suggest that C9orf72 may regulate motor neuron survival through regulation of inflammatory responses by affecting autophagy-lysosomal function of microglia and macrophages. In contrast, our studies failed to detect any obvious abnormalities of microglia in our C9orf72 deficient mice (Fig. 9). It is possible that this may simply be due to our mice not being old enough (10 months old) for us to detect a microglial phenotype. It is also likely that microglial abnormalities may become apparent in the C9orf72 deficient mice upon additional insults to the nervous system.

The physical interaction between the C9orf72/SMCR8/WDR41 complex and the autophagy initiation complex FIP200/Ulk1/ATG13/ATG101 (Fig. 4) suggests that C9orf72/SMCR8/WDR41 might regulate autophagy through the FIP200/Ulk1/ATG13/ATG101 complex. However, exactly how C9orf72/SMCR8/WDR41 regulates autophagy remains unclear. It is interesting that WDR41 is enriched at the Golgi, as the ER-Golgi intermediate compartment has been shown to serve as a key membrane source for autophagosome biogenesis [16]. One possibility is that C9orf72/SMCR8/WDR41 serves as a substrate and a downstream signaling component after Ulk1 activation. Interestingly, SMCR8 was shown to be a substrate for Ulk1 in a recent study [41]. This observation together with reduced autophagy initiation in C9or72-/- spleen tissues argues that C9orf72/SMCR8/WDR41 functions downstream of Ulk1 activation during autophagy activation. Autophagy defects in C9orf72-/- mice might trigger upregulation of autophay/lysosome genes to compensate for the defect. Although we failed to identify any Rab GTPases as C9orf72 interactors in our proteomic screen, C9orf72/SMCR8/WDR41 was shown to interact with Rab8a and Rab39b and functions as a guanine nucleotide exchange factor (GEF) for Rab GTPases in a recent study [41]. However, the effect of Ulk1 phosphorylation on the GEF activities remains to be determined. Future endeavors are needed to further understand the mechanistic functions and regulations of C9orf72/SMCR8/WDR41 complex at molecular and cellular levels. More studies are also needed to characterize neuronal and microglial phenotypes due to C9orf72 loss, possibly upon additional challenges, to explain how C9orf72 deficiency contributes to ALS/FTLD progression.

## Conclusions

We describe the identification of two binding partners for C9orf72: SMCR8 and WDR41. We demonstrated that C9orf72/SMCR8/WDR41 interacts with the FIP200/Ulk1/ATG13/ATG101 complex. We also generated C9orf72 deficient mice and showed that loss of C9orf72 leads to macrophage infiltration in multiple organs. Additionally, C9orf72 deficiency leads to autophagy defects and increased levels of many lysosomal proteins, supporting a critical role of C9orf72 in regulating autophagy/lysosomal pathway and inflammation in vivo.

### Ethical approval

All applicable international, national, and/or institutional guidelines for the care and use of animals were followed.

## Additional files

Additional file 1: Figure S1. Levels of endogenous versus overexpressed C9orf72 in N2a and HEK293T cells. N2a (a) and HEK293T (b) cells were transfected with GFP control or GFP-C9orf72. Cells were lysed 2 days after transfection and lysates were subjected to Western blot using anti-C9orf72 antibodies (Proteintech). (PDF 146 kb) Additional file 2: Table S1. List of hits from the SILAC proteomic screen. Proteins with more than 10 peptides and Log2 fold changes (GFP/GFP-C9orf72) < -1.00 are listed. Count = total number of peptides identified; count-h: peptides in the heavy fraction; count-l: peptides in the light fraction. (PDF 155 kb) Additional file 3: Figure S2. Absence of C9orf72 isoform I in the C9orf72 CRISPR targeted mice. a. Western blot of brain lysates from WT or C9orf72 deficient (KO) mice using various C9orf72 antibodies as indicated. b. Western blot analysis of C9orf72 protein levels in wild type (WT) and C9orf72 knockout (KO) mouse tissues with anti-C9-L antibodies. (PDF 147 kb) Additional file 4: Figure S3. C9orf72 deficiency in mice leads to age dependent spleen enlargement. Representative images of spleen dissected from 2 month, 5 month and 10 month old WT and C9or72-/- mice are shown. Scale bar = 1 cm. (PDF 217 kb)
