# Supplementary material for: The ALS/FTLD associated protein C9orf72 associates with SMCR8 and WDR41 to regulate the autophagy-lysosome pathway
Source: Acta Neuropathol Commun. 2016 May 18;4:51. doi: 10.1186/s40478-016-0324-5 (PMC4870812; doi:10.1186/s40478-016-0324-5)
Supplement: Additional file 1: Figure S1. — Levels of endogenous versus overexpressed C9orf72 in N2a and HEK293T cells. N2a (a) and HEK293T (b) cells were transfected with GFP control or GFP-C9orf72. Cells were lysed 2 days after transfection and lysates were subjected to Western blot using anti-C9orf72 antibodies (Proteintech). (PDF 146 kb) [file 40478_2016_324_MOESM1_ESM.pdf]

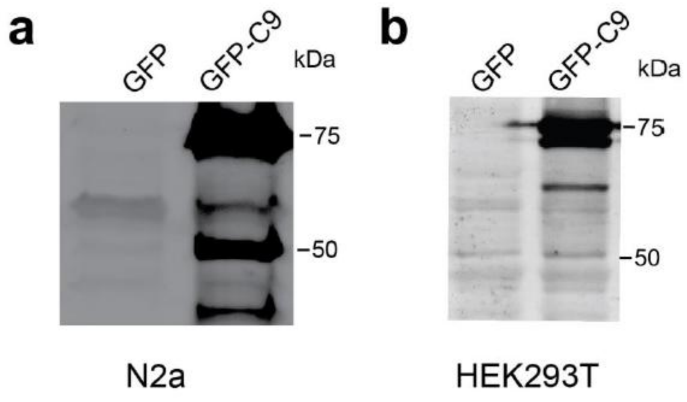

Figure S1: Levels of endogenous versus overexpressed C9orf72 in N2a and HEK293T cells. N2a (a) and HEK293T (b) cells were transfected with GFP control or GFP-C9orf72. Cells were lysed 2 days after transfection and lysates were subjected to Western blot using anti-C9orf72 antibodies (Proteintech) to determine the levels of endogenous vs overexpressed C9orf72.
