# Supplementary material for: The ALS/FTLD associated protein C9orf72 associates with SMCR8 and WDR41 to regulate the autophagy-lysosome pathway
Source: Acta Neuropathol Commun. 2016 May 18;4:51. doi: 10.1186/s40478-016-0324-5 (PMC4870812; doi:10.1186/s40478-016-0324-5)
Supplement: Additional file 2: Table S1. — List of hits from the SILAC proteomic screen. Proteins with more than 10 peptides and Log2 fold changes (GFP/GFP-C9orf72) < -1.00 are listed. Count = total number of peptides identified; count-h: peptides in the heavy fraction; count-l: peptides in the light fraction. (PDF 155 kb) [file 40478_2016_324_MOESM2_ESM.pdf]

Protein	Log2 fold change (GFP/GFP-C9 IP)	P Value	count	count_H	count_L
WDR41	-5.43072	1.85E-07	23	22	1
3110043O21RIK	-4.55343	1.58E-07	144	114	30
SMCR8	-4.16577	1.75E-06	64	57	7
VDAC3	-3.5849	3.89E-07	29	25	4
SLC25A4	-2.51662	3.87E-06	20	15	5
SLC25A5	-2.48505	5.23E-06	50	33	17
CHCHD3	-2.15049	8.81E-05	14	11	3
SLC25A12	-2.11025	1.64E-06	29	22	7
IMMT	-2.08775	4.91E-05	27	23	4
SLC3A2	-1.6362	6.02E-06	16	11	5
SLC25A1	-1.59634	3.73E-06	17	13	4
LOC100046151	-1.54033	7.94E-06	14	7	7
FAR1	-1.51678	9.92E-05	14	11	3
SLC25A11	-1.51539	4.62E-06	11	8	3
DNAJA1	-1.4873	1.15E-05	38	29	9
GCN1L1	-1.41411	0.000418	18	16	2
DNAJB11	-1.38731	1.82E-05	14	9	5
DNAJA2	-1.38438	2.42E-05	27	21	6
KARS	-1.31829	0.003613	13	10	3
ASS1	-1.2955	0.0006	13	10	3
TUBA4A	-1.25374	4.6E-05	11	6	5
SLC25A4	-1.24254	3.49E-05	59	34	25
MTAP1B	-1.18204	9.49E-05	79	57	22
SLC25A4	-1.10584	0.000127	24	13	11
NSF	-1.0949	0.00141	11	7	4
2310079N02RIK	-1.01951	0.000344	15	10	5
TUBA1A	-1.00626	0.000273	30	17	13

Table S1: List of hits from the SILAC proteomic screen.

Proteins with more than 10 peptides and Log2 fold changes (GFP/GFP-C9orf72) <-1.00 are listed

count=total number of peptides identified;

count_H: number of peptides in the heavy fraction;

count_L: number of peptides in the light fraction.
