# Supplementary material for: The ALS/FTLD associated protein C9orf72 associates with SMCR8 and WDR41 to regulate the autophagy-lysosome pathway
Source: Acta Neuropathol Commun. 2016 May 18;4:51. doi: 10.1186/s40478-016-0324-5 (PMC4870812; doi:10.1186/s40478-016-0324-5)
Supplement: Additional file 3: Figure S2. — Absence of C9orf72 isoform I in the C9orf72 CRISPR targeted mice. a. Western blot of brain lysates from WT or C9orf72 deficient (KO) mice using various C9orf72 antibodies as indicated. b. Western blot analysis of C9orf72 protein levels in wild type (WT) and C9orf72 knockout (KO) mouse tissues with anti-C9-L antibodies. (PDF 147 kb) [file 40478_2016_324_MOESM3_ESM.pdf]

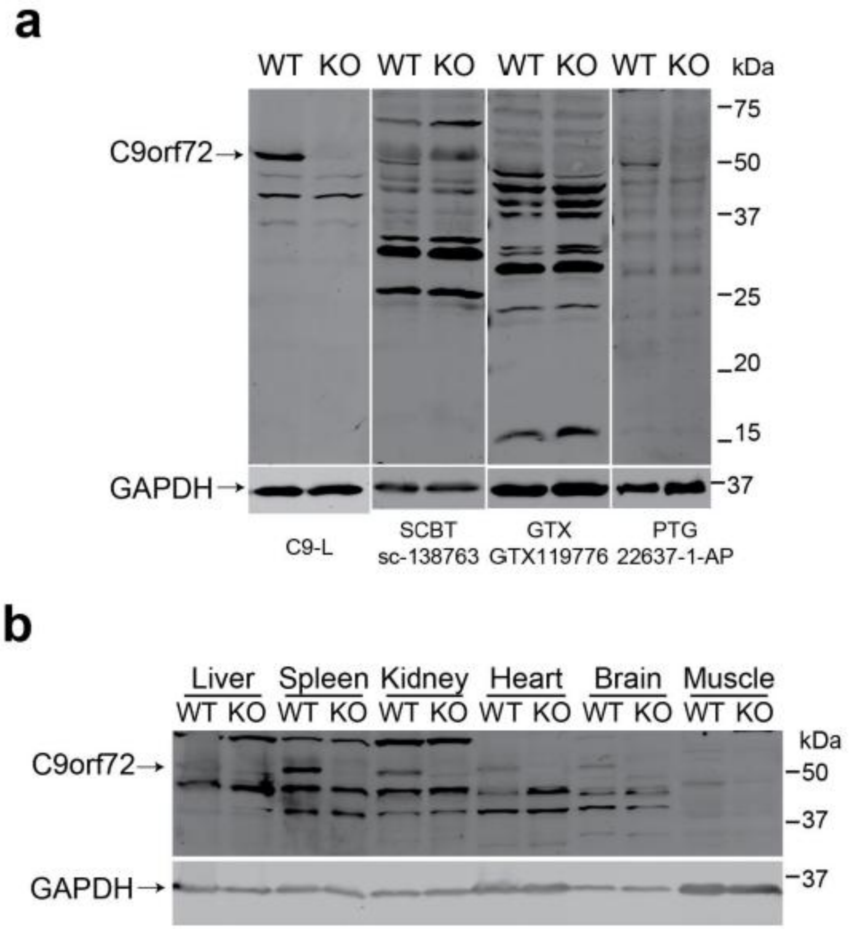

Figure S2: Absence of C9orf72 isoform I in the C9orf72 CRISPR targeted mice. a. Western blot of brain lysates from WT or C9orf72 deficient (KO) mice using various C9orf72 antibodies as indicated. **b.** Western blot analysis of C9orf72 protein levels in wild type (WT) and C9orf72 knockout (KO) mouse tissues with anti-C9-L antibodies.
