# Supplementary material for: The ALS/FTLD associated protein C9orf72 associates with SMCR8 and WDR41 to regulate the autophagy-lysosome pathway
Source: Acta Neuropathol Commun. 2016 May 18;4:51. doi: 10.1186/s40478-016-0324-5 (PMC4870812; doi:10.1186/s40478-016-0324-5)
Supplement: Additional file 4: Figure S3. — C9orf72 deficiency in mice leads to age dependent spleen enlargement. Representative images of spleen dissected from 2 month, 5 month and 10 month old WT and C9or72-/- mice are shown. Scale bar = 1 cm. (PDF 217 kb) [file 40478_2016_324_MOESM4_ESM.pdf]

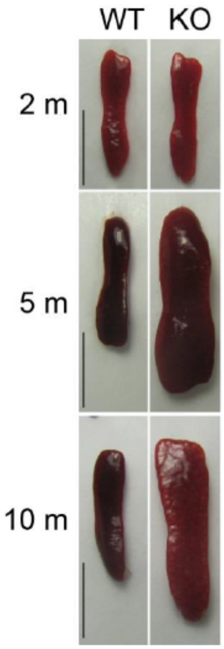

Figure S3: C9orf72 deficiency in mice leads to age dependent spleen enlargement. Representative images of spleen dissected from 2 month, 5 month and 10 month old WT and C9or72-/- mice are shown. Scale bar=1cm.
